# Reconsideration of Amyloid Hypothesis and Tau Hypothesis in Alzheimer's Disease

**DOI:** 10.3389/fnins.2018.00025

**Published:** 2018-01-30

**Authors:** Fuyuki Kametani, Masato Hasegawa

**Affiliations:** Department of Dementia and Higher Brain Function, Tokyo Metropolitan Institute of Medical Science, Tokyo, Japan

**Keywords:** Alzheimer's disease, Aβ, APP, amyloid, tau, PHF

## Abstract

The so-called amyloid hypothesis, that the accumulation and deposition of oligomeric or fibrillar amyloid β (Aβ) peptide is the primary cause of Alzheimer's disease (AD), has been the mainstream concept underlying AD research for over 20 years. However, all attempts to develop Aβ-targeting drugs to treat AD have ended in failure. Here, we review recent findings indicating that the main factor underlying the development and progression of AD is tau, not Aβ, and we describe the deficiencies of the amyloid hypothesis that have supported the emergence of this idea.

## Introduction

Alzheimer's disease (AD) is said to account for about 70% of dementia. The affected brain exhibits astroglyosis, nerve cell atrophy and neuronal loss, and is characterized by the extensive distribution of two kinds of abnormal structures: so-called senile plaques and neurofibrillary tangles (NFTs). In the 1980's, it was shown that senile plaque consists of amyloid fibrils composed of the amyloid β (Aβ) peptide (Glenner and Wong, 1984; Masters et al., 1985), while NFT contain bundles of paired helical filaments of the microtubule-associated protein tau by immunochemically (Brion et al., 1985; Grundke-Iqbal et al., 1986; Nukina and Ihara, 1986) and biochemically (Goedert et al., 1988; Kondo et al., 1988; Kosik et al., 1988; Wischik et al., 1988b).

**Figure 1:**
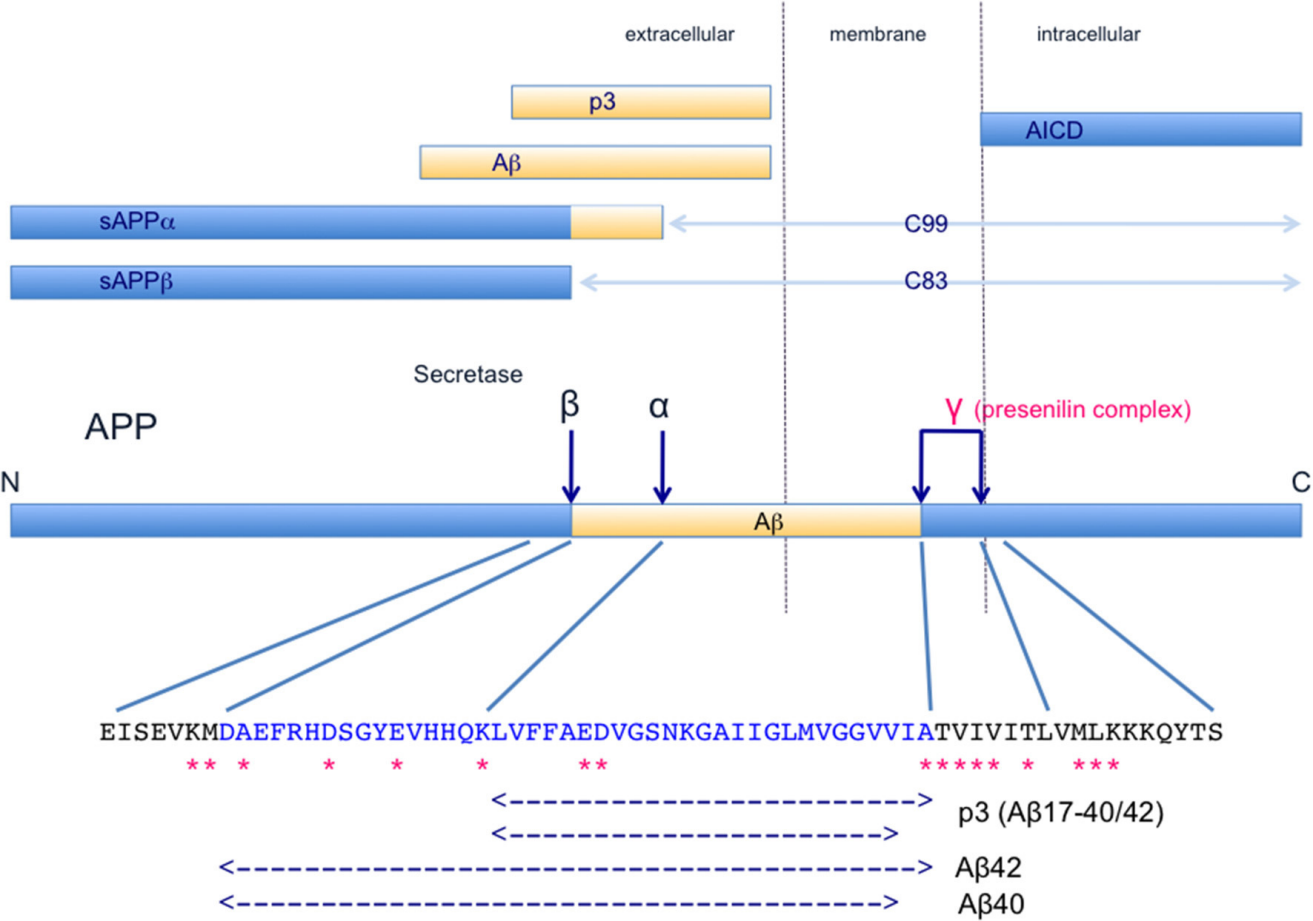
Schematic illustration of the structure and metabolism of APP and its derivatives. Dark blue arrows indicate cleavage sites. α-Secretase (TACE/ADAM) (Buxbaum et al., 1998; Lammich et al., 1999) cleaves the α-site and β-secretase (BACE1, Vassar et al., 1999) cleaves the β-site, affording N-terminal fragments, sAPPα and sAPP β, and C-terminal fragments, C83 and C99, respectively. C83 and C99 are further cleaved at the γ-sites by γ-secretase complex, which includes presenilin-1, nicastrin, Aph-1 and Pen2 (Capell et al., 1998; De Strooper et al., 1998; Yu et al., 2000; Francis et al., 2002; Goutte et al., 2002; Takasugi et al., 2003). AICD and p3/Aβ are produced and released from the membrane. In the normal physiological state, α-secretase cleaves 90% or more of APP and the remaining APP is cleaved by β-secretase. Therefore, the major products in this APP metabolic pathway are sAPPα, C83, p3, and AICD, and Aβ is a minor product. AICD is rapidly degraded (Cupers et al., 2001; Kopan and Ilagan, 2004; Kametani and Haga, 2015). Thus, mutations found in familial AD, especially presenilin mutations, may affect the formation and processing of a variety of products. A part of APP sequence including Aβ is shown. Asterisks indicate APP mutations that have been identified in familial AD. These pathogenetic mutations of APP cluster near the α-secretase, β-secretase and γ-secretase cleavage sites. These mutations cause accumulation of APP C-terminal fragments (Tesco et al., 2005; Wiley et al., 2005; Xu et al., 2016a), and such accumulation has been found even in sporadic AD brains (Pera et al., 2013). Furthermore, mutations in presenilin, a constitutive protein of the γ-secretase complex, reduce γ-secretase activity (Chen et al., 2002; Walker et al., 2005; Bentahir et al., 2006; Shen and Kelleher, 2007; Xia et al., 2015). Decrease in the catalytic capacity of γ-secretase, which would lead to an increase of APP C-terminal fragments, facilitates the pathogenesis in sporadic and familial AD (Svedruzic et al., 2015).

Aβ is a peptide consisting of about 40 amino acids, formed by sequential cleavages of amyloid β precursor protein (APP, http://www.uniprot.org/uniprot/P05067) by β-secretase (BACE 1) and γ-secretase (a complex containing presenilin 1), as illustrated in Figure 1. APP is a transmembrane protein associated with neuronal development, neurite outgrowth, and axonal transport (Kang et al., 1987). On the other hand, tau is a microtubule-associated protein that promotes microtubule polymerization and stabilization, and the abilities are regulated by phosphorylation (http://www.uniprot.org/uniprot/P10636).

Studies of AD pathogenesis have mostly been focused on how Aβ and tau form senile plaques and NFTs, respectively, and how these abnormal structures induce neural degeneration and neuronal loss.

## The amyloid hypothesis

The amyloid hypothesis (also known as the amyloid cascade hypothesis, the Aβ hypothesis, etc.) has been the mainstream explanation for the pathogenesis of AD for over 25 years (Hardy and Allsop, 1991; Selkoe, 1991; Hardy and Higgins, 1992; Hardy and Selkoe, 2002), and may be briefly summarized as follows (Figure 1). In normal subjects, Aβ is excised from APP by β- and γ-secretase and released outside the cell, where it is rapidly degraded or removed. However, in aged subjects or under pathological conditions, the metabolic ability to degrade Aβ is decreased, and Aβ peptides may be accumulated. Aβ 40 and Aβ 42 (more hydrophobic than Aβ 40), containing 40 and 42 amino acid residues, respectively, are major components of the accumulated Aβ (Figure 1). An increase in the level of Aβ 42 or an increase in the ratio of Aβ 42 induces Aβ amyloid fibril formation, and the accumulated Aβ amyloid fibrils develop into senile plaque, causing neurotoxicity and induction of tau pathology, leading to neuronal cell death and neurodegeneration.

The APP gene is on chromosome 21 (Kang et al., 1987), and the discovery of genetic mutations of APP in early-onset familial AD (http://www.alzforum.org/mutations), as shown in Figure 1, appeared to support the amyloid hypothesis. These pathogenetic mutations of APP are clustered near β-secretase or γ-secretase cleavage sites, and are associated with an increase in Aβ42 production and/or a change in the ratio of Aβ42 formation. Interestingly, Down's syndrome patients with trisomy 21 exhibit AD-like pathology by about 40 years of age (Kolata, 1985), and this was thought to be due to the fact that the amount of APP in the brain was increased to 1.5 times the normal amount and the amount of Aβ was also increased (Kolata, 1985). In addition, APP locus duplication causes autosomal-dominant early-onset AD with cerebral amyloid angiopathy, with accumulation of large amounts of Aβ peptides (Delabar et al., 1987; Rovelet-Lecrux et al., 2006). Moreover, other familial AD mutations have been identified in presenilin 1/2, which is a component of γ-secretase (http://www.alzforum.org/mutations) (Figure 1). These mutations in APP and presenilin are closely linked to the Aβ production process, providing a rational basis for the idea that Aβ production and/or Aβ amyloid fibril formation represent the central pathogenic cause of AD (Hardy and Allsop, 1991; Selkoe, 1991; Hardy and Higgins, 1992; Hardy and Selkoe, 2002).

## Problems with the amyloid hypothesis

To investigate the pathogenesis of AD, a number of genetically modified mouse models were produced in which Aβ is deposited in the brain. However, although senile plaques (accumulation of Aβ amyloid fibrils) are formed in these mice, NFT formation (accumulation of tau) and nerve cell death have not been observed (Bryan et al., 2009) (http://www.alzforum.org/research-models/alzheimers-disease). This suggested that extracellular accumulation of Aβ fibrils is not intrinsically cytotoxic, and also that Aβ does not induce tau accumulation. As Aβ is a normal metabolic product of APP and is not itself toxic under normal physiological conditions, the idea developed that Aβ oligomers (multimers) were the key toxic agents.

It has been reported that synaptic failures occur from an early stage in the AD brain and that the levels of synaptic proteins change (Masliah et al., 2001). Also, a drastic decrease in the number of synapses is characteristically observed in AD (Davies et al., 1987). Therefore, it was suggested that Aβ, which is present abundantly at an early stage after birth in AD model mice, causes synaptic impairment (William et al., 2012). Furthermore, it was reported that decrease of dendritic spines, inhibition of long-term potentiation, promotion of long-term suppression, and impairment of memory learning occur when Aβ oligomers (dimers) obtained from AD patients' brains were directly transferred to hippocampus of mouse brain (Shankar et al., 2008). However, although Aβ oligomers and Aβ amyloid fibrils were present in Aβ42-overexpressing BRI2-Aβ mice, and amyloid deposits and formation of senile plaques were observed in the brain, degeneration of nerve cells and neuronal loss were not observed, and there was no impairment of cognitive functions (Kim et al., 2007, 2013). These results indicate that Aβ42, including its oligomers and amyloid fibrils, is not cytotoxic. In addition, various immunotherapies targeting Aβ in AD model mice were effective in decreasing Aβ deposition in the brains, but it did not lead to improvement of actual symptoms or accumulation of tau (Ostrowitzki et al., 2012; Giacobini and Gold, 2013; Doody et al., 2014; Salloway et al., 2014).

Recent advances in amyloid imaging have made it possible to observe Aβ amyloid accumulation in the patient's brain. As a result, it has been found that there are many normal patients with amyloid deposits, and also AD patients with very few amyloid deposits (Edison et al., 2007; Li et al., 2008). Further, in the brain of elderly non-demented patients, the distribution of senile plaques is sometimes as extensive as that of dementia patients (Davis et al., 1999; Fagan et al., 2009; Price et al., 2009; Chetelat et al., 2013). This suggests that Aβ amyloid deposition is a phenomenon of aging, and has no direct relation with the onset of AD.

Taking these facts into account, it appears that neurodegeneration/neuronal loss and amyloid deposition are independent, unrelated phenomena (Chetelat, 2013), contrary to the amyloid hypothesis.

## Reconsideration of APP and presenilin (PS) mutations in familial AD

In this section, we will focus on the nature and effects of mutations that are reported to be associated with familial AD.

In the normal physiological state, α-secretase cleaves 90% or more of APP and the remaining APP is cleaved by β-secretase, then γ-secretase cleaves the C-terminal region, as shown in Figure 1. The major products of this APP metabolic pathway are sAPPα, C83, p3, and APP intracellular domain (AICD), and Aβ is a minor product. Moreover, AICD is rapidly degraded (Cupers et al., 2001; Kopan and Ilagan, 2004; Kametani and Haga, 2015), suggesting that APP C-terminal fragments (C83, C99, and AICD) may be toxic and need to be removed (Kametani, 2008; Robakis and Georgakopoulos, 2014). Thus, when considering the effects of familial AD mutations, the effects on all the major products of APP metabolism should be considered.

AD-associated mutations in PS (PS1), a constituent protein of the γ-secretase complex, reduce γ-secretase activity, leading to decreased production of Aβ, especially Aβ40 (Chen et al., 2002; Walker et al., 2005; Bentahir et al., 2006; Shen and Kelleher, 2007; Xia et al., 2015). But, as a result, the proportion of Aβ42 increases and Aβ amyloid is formed. At the same time, APP C-terminal fragments that should be cleaved by γ-secretase are not cleaved, and accumulate in the cell membrane (Chen et al., 2002; Kametani, 2008; Robakis and Georgakopoulos, 2014).

It has also been reported that APP mutation causes accumulation of APP C-terminal fragments (Tesco et al., 2005, p.181; Wiley et al., 2005, p. 205; Xu et al., 2016a) and that the production of APP C-terminal fragment by β-secretase increases in the brain of patients with sporadic AD (Pera et al., 2013). Moreover, decrease in the catalytic capacity of γ-secretase, which would promote accumulation of APP C-terminal fragments, might facilitate the development of both sporadic and familial AD with APP mutation (Svedruzic et al., 2015).

Further, γ-secretase inhibitor may accelerate accumulation of APP C-terminal fragments in brain, if it is used as a therapeutic agent to suppress Aβ production in AD patients. Notably, the symptoms of AD worsened in a clinical trial of γ-secretase inhibitor (Doody et al., 2013).

These indicate that APP C-terminal fragment accumulation closely links to pathogenesis of sporadic and familial AD.

It was previously reported that APP or APP fragments accumulated in dystrophic neurites in AD brains (Ishii et al., 1989) and that the accumulation of APP and its metabolic fragments induced neurotoxicity and vesicular trafficking impairment (Yoshikawa et al., 1992; Kametani et al., 2004; Roy et al., 2005). It has also been reported that synaptic disorders and dendritic dysplasia occur in the absence of Aβ amyloid deposition (Boncristiano et al., 2005), and that C-terminal fragments of APP cause synaptic failure and memory impairment (Tamayev et al., 2012). Furthermore, transgenic mice expressing the C-terminal intracellular domain of APP (AICD) developed Alzheimer's-like symptoms, such as accumulation of phosphorylated tau and memory impairment (Ghosal et al., 2009). Also, accumulation of APP C-terminal fragments triggers the hydrolysis of cAMP, causing impairment of the cAMP/PKA/CREB pathway (Kametani and Haga, 2015). Moreover, APP C-terminal fragment accumulation alters the subcellular localization of APP and the distribution of Rab11, and decreases endocytosis and soma-to-axon transcytosis of LDL (Woodruff et al., 2016), and this affects axonal vesicle trafficking (Szpankowski et al., 2012; Fu and Holzbaur, 2013; Gunawardena et al., 2013). These findings support the idea that APP C-terminal fragment accumulation causes neuronal impairment.

In addition, sAPPα is involved in neurite outgrowth and has a neuroprotective effect (Baratchi et al., 2012), and AICD is involved in signal transduction (Cao and Sudhof, 2001). Thus, multiple APP domains, including the C-terminus, are required for normal nervous system function (Klevanski et al., 2015). Therefore, since APP metabolites play a variety of functions in the brain, impaired APP metabolism may have a range of effects.

Overall, these findings suggest that the trigger of AD are closely linked to impairments of APP metabolism and accumulation of APP C-terminal fragments, rather than Aβ production and Aβ amyloid formation.

## The tau hypothesis

**Figure 2:**
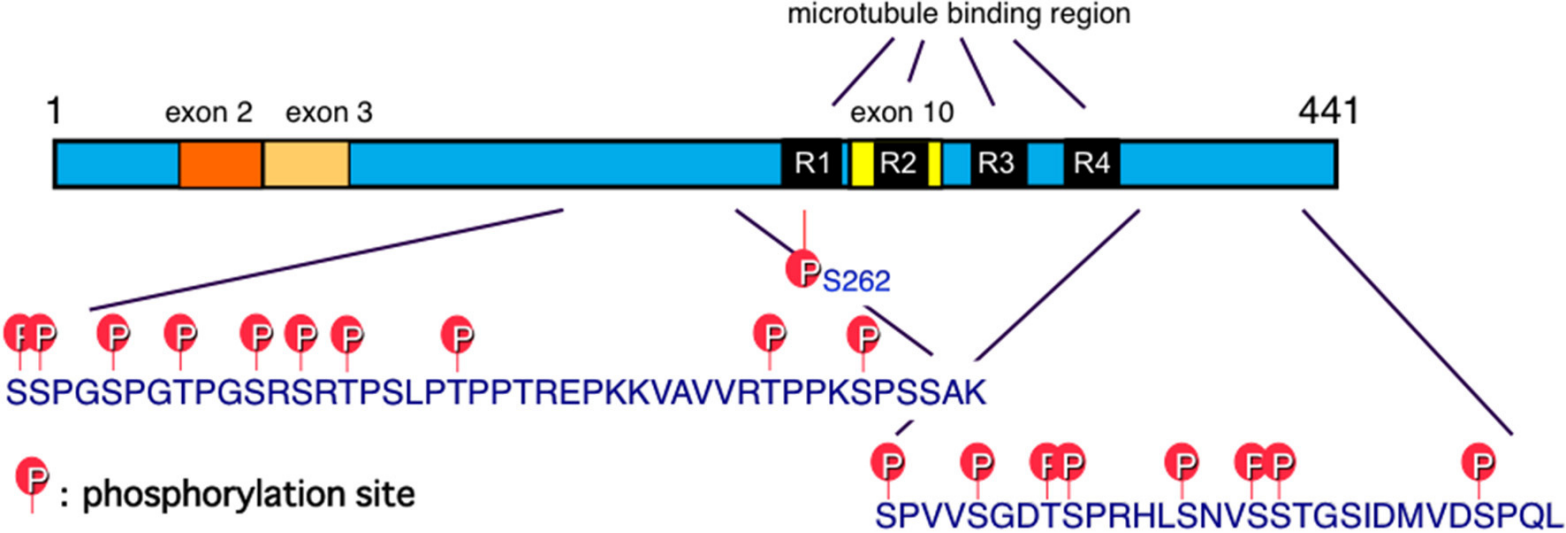
Schematic illustration of functional sites of tau. Six tau isoforms are expressed in the adult human brain as a result of mRNA alternative splicing, with or without exons 2, 3, and 10. Exon 10 contains the microtubule-binding region. Insertion of exon 10 affords 4-repeat (4R) tau isoforms, while 3-repeat (3R) tau isoforms are produced without exon 10 (Goedert et al., 1989a). Major tau phosphorylation sites identified in PHF-tau from AD brains are shown. Microtubule binding regions R3 and R4 form the core of tau fibrils (PHF and SF) (Taniguchi-Watanabe et al., 2016; Fitzpatrick et al., 2017).

Tau is one of the microtubule-associated proteins that regulate the stability of tubulin assemblies. The human tau gene is localized in chromosome 17. Six tau isoforms are expressed in the adult human brain as a result of mRNA alternative splicing, with or without exons 2, 3, and 10 (Goedert et al., 1989a; Figure 2). Exon 10 contains the microtubule-binding region. Insertion of exon 10 affords 4-repeat (4R) tau isoforms, while 3-repeat (3R) tau isoforms are produced without exon 10 (Figure 2). Adult human brain expresses both 3R and 4R tau isoforms, which are located mainly in axons of adult neurons under normal physiological conditions. The tau hypothesis is that the principle causative substance of AD is tau.

In AD brains, 3R and 4R tau is accumulated in a hyperphosphorylated state in the pathological inclusions (Goedert, 1993; Goedert et al., 1996; Serrano-Pozo et al., 2011; Iqbal et al., 2016). Ultrastructurally, unique twisted fibrils with ~80 nm periodicity appearing as paired helical filaments (PHFs) or related straight filaments (SFs) are observed (Crowther and Wischik, 1985; Wischik et al., 1988a,b; Crowther et al., 1989; Goedert et al., 1989b; Greenberg and Davies, 1990; Lee et al., 1991). These pathological inclusions are referred to as neurofibrillary tangles (NFTs) if they are formed in neuronal cell bodies, while they are referred to as threads if they are formed in dendrites or axons. These findings suggested that mis-sorting of tau might induce tau pathology (Zempel and Mandelkow, 2014).

Tau pathology is staged according to Braak and Braak (Braak and Braak, 1991), and appears first in the transentorhinal region (stages I and II), then spreads to the limbic region (stages III and IV) and neocortical areas (stages V and IV). This spreading of tau pathology is strongly correlated with the extent of cognitive and clinical symptoms. Recent PET studies have shown that the spatial patterns of tau tracer binding are closely linked to the patterns of neurodegeneration and the clinical presentation in AD patients (Bejanin et al., 2017; Okamura and Yanai, 2017) and that subjective cognitive decline is indicative of early tauopathy in the medial temporal lobe, specifically in the entorhinal cortex, and to a lesser extent with elevated global levels of Aβ (Scholl et al., 2016; Schwarz et al., 2016; Buckley et al., 2017). Furthermore, it has been reported that tau lesions occurred earlier than Aβ accumulation (Braak and Del Tredici, 2014; Johnson et al., 2015). Thus, progression of AD is strongly associated with tau pathology, rather than Aβ amyloid accumulation.

Tau pathologies are also seen in other neurodegenerative dementing disorders, such as frontotemporal dementia and parkinsonism linked to chromosome 17 (FTDP-17), Pick's disease (PiD), progressive supranuclear palsy (PSP), corticobasal degeneration (CBD), argyrophilic grain disease (AGD), tangle-only dementia, and chronic traumatic encephalopathies (CTE) (Iwatsubo et al., 1994; Spillantini et al., 1997, 1998; Hutton et al., 1998; Poorkaj et al., 1998; Buee and Delacourte, 1999; Goedert and Hasegawa, 1999; Lee et al., 2001; Kovacs, 2015) In particular, FTDP-17 patients exhibit many exonic and intronic mutations in the tau gene (http://www.alzforum.org/mutations), resulting in tau accumulation (Spillantini et al., 1997, 1998; Hutton et al., 1998; Poorkaj et al., 1998). These findings suggest that tau abnormalities cause accumulation of tau and degeneration of neurons. In other sporadic cases of tauopathies, including AD, the initial trigger is unclear, but wild-type tau is accumulated.

What is the tau-induced neurodegeneration? In FTDP-17, the disease causing tau-mutations cluster near the C-terminal microtubule binding repeat and impair the ability of tau to bind microtubules (Hasegawa et al., 1998), suggesting impairment of the microtubule regulation. Mis-localized tau also induces impairment of microtubule regulation (Zempel and Mandelkow, 2014). These taus form aggregation and fibril seed, and were hyperphosphorylated as described above. Furthermore, the stability of mutant and hyperphosphorylated tau increases compared to the normal tau (Yamada et al., 2015; Bardai et al., 2018). Aberrant interaction of stabilized tau with filamentous actin induces mis-stabilization of actin (Fulga et al., 2007), synaptic impairment (Cabrales Fontela et al., 2017; Zhou et al., 2017; Bardai et al., 2018), and defects in mitochondrial integrity (DuBoff et al., 2012). Therefore, tau pathology causes extensive damage in the cell, such as transport system, cytoskeletal system, signaling system, and mitochondrial integrity.

## Propagation of tau pathology

In an experimental model of cultured cells and mice, abnormal tau (amyloid-like fibril tau) converts normal tau to an abnormal type. Therefore, it has been hypothesized that tau aggregates form first in a small number of brain cells, from where they propagate to other regions, resulting in neurodegeneration and disease. This hypothesis has recently gained attention because it has been confirmed that tau proliferates and propagates between cells (Clavaguera et al., 2009, 2013; Nonaka et al., 2010; Hasegawa, 2016; Goedert and Spillantini, 2017). The existence of several human tauopathies with distinct fibril morphologies has led to the suggestion that different molecular conformers (or strains) of aggregated tau exist (Goedert and Spillantini, 2017). Although the transmission mechanism of tau aggregates from cell to cell is still not clear, tau pathology does spread in the brain in a well-defined manner; its distribution can be correlated with the clinical stages of disease (Braak and Braak, 1991), and it is considered that tau pathology correlates better than Aβ pathology with clinical features of dementia. Recently, we found that increase APP with or without familial AD mutations, not Aβ, may work as a receptor of abnormal tau fibrils and promote intracellular tau aggregation (Takahashi et al., 2015), suggesting that APP rather than Aβ may accelerate tau accumulation and propagation.

## AD risk factors, ApoE4 and TREM2

Apolipoprotein E (ApoE) is one of the major apolipoproteins (http://www.uniprot.org/uniprot/P02649). The *ApoE* gene has three alleles, ε*2*, ε*3*, and ε*4*, corresponding to isoforms E2, E3, and E4, respectively. In the central nervous system, ApoE produced and secreted by astrocytes and microglia binds to lipoprotein and is taken up into nerve cells via the ApoE receptor during the developmental stage of the central nervous system and the repair period after neuronal damage.

The *ApoE4* allele is a genetic risk factor for sporadic AD (Corder et al., 1993). As the number of ε*4* genes increases, the age of onset of AD declines and the incidence of AD increases (Maestre et al., 1995), and there is an increased risk of 3–4 and 8–12 times for one or two copies of the allele, respectively. It is considered that impaired apoE4 function affects the clearance pathway of Aβ (Zlokovic, 2013; Robert et al., 2017) and modulates Aβ-induced effects on inflammatory receptor signaling, including amplification of detrimental pathways and suppression of beneficial pathways (Chan et al., 2015; Tai et al., 2015). To examine the role of ApoE, human ApoE targeted replacement mice were crossed with mutant human amyloid precursor protein (APP) mice. In this context, ApoE genotypes only modulate Aβ-mediated insulin signaling impairment (Chan et al., 2015). Recently, however, P301S tau transgenic mice were generated on either a human ApoE knock-in (KI) or ApoE knockout (KO) background, and developed significant brain atrophy primarily in the hippocampus, piriform/entorhinal cortex, and amygdala, accompanied by significant lateral ventricular enlargement (Shi et al., 2017). ApoE plays an important role in regulating tau-mediated neurodegeneration and neuroinflammation, with ApoE4 causing more severe damage and the absence of ApoE being protective (Shi et al., 2017). These findings indicate that ApoE4 affects neurodegeneration independently of Aβ and Aβ amyloid in the context of tau pathology (Shi et al., 2017).

Triggering receptor expressed on myeloid cells 2 (TREM2) is expressed on the membranes of microglia and is critical for the response to injury and AD pathology (http://www.uniprot.org/uniprot/Q9NZC2). TREM2 recognizes lipoproteins including ApoE, phospholipid and apoptotic cells and is implicated in microglial phagocytosis. Variants in the *TREM2* gene increase the risk of getting AD. Initially, this was thought to be related to the elimination of Aβ plaque. TREM2 deficiency in the setting of pure tauopathy limits gliosis and neuroinflammation, as well as protecting against brain atrophy, suggesting that TREM2 facilitates a microglial response to tau pathology and/or tau-mediated damage in the brain (Bemiller et al., 2017; Leyns et al., 2017). These results are consistent with the findings of strikingly reduced inflammation and neurodegeneration in mice lacking ApoE, as described above. Therefore, the TREM2-ApoE pathway is important for facilitating the microglial response to damage in the brain, and a functional consequence of activation of the TREM2-ApoE pathway is that microglia lose the ability to regulate brain homeostasis (Krasemann et al., 2017; Ulland et al., 2017). Microglial inflammation promotes tau-dependent degeneration independently of Aβ and Aβ amyloid.

## APP trigger tauopathy

APP turn over rapidly and easily metabolize (Oltersdorf et al., 1990; Weidemann et al., 2002). Therefore, impairment of APP metabolism has a serious effect on cells. Increased APP and/or its C-terminal fragments induce axonal and synaptic defects (Rusu et al., 2007; Rodrigues et al., 2012; Deyts et al., 2016; Xu et al., 2016b), thereby triggering the mis-localization of tau (Blurton-Jones and Laferla, 2006; Hochgrafe et al., 2013). This protein modulates motility in a motor-specific manner to direct intracellular transport (Chaudhary et al., 2017). Mis-localized tau proteins accumulate, form fibril seeds and propagate (He et al., 2017). The pathological tau induce further transport dysfunction (Goldsbury et al., 2006; Rusu et al., 2007), creating a vicious circle and leading to tau accumulation. Moreover, since overexpression of APP promoted the seed aggregation of intracellular tau in cultured cell, suggesting that APP may function as a receptor of abnormal tau fibrils (Takahashi et al., 2015). Thus, increased APP may accelerate pathological incorporation and propagation.

**Figure 3:**
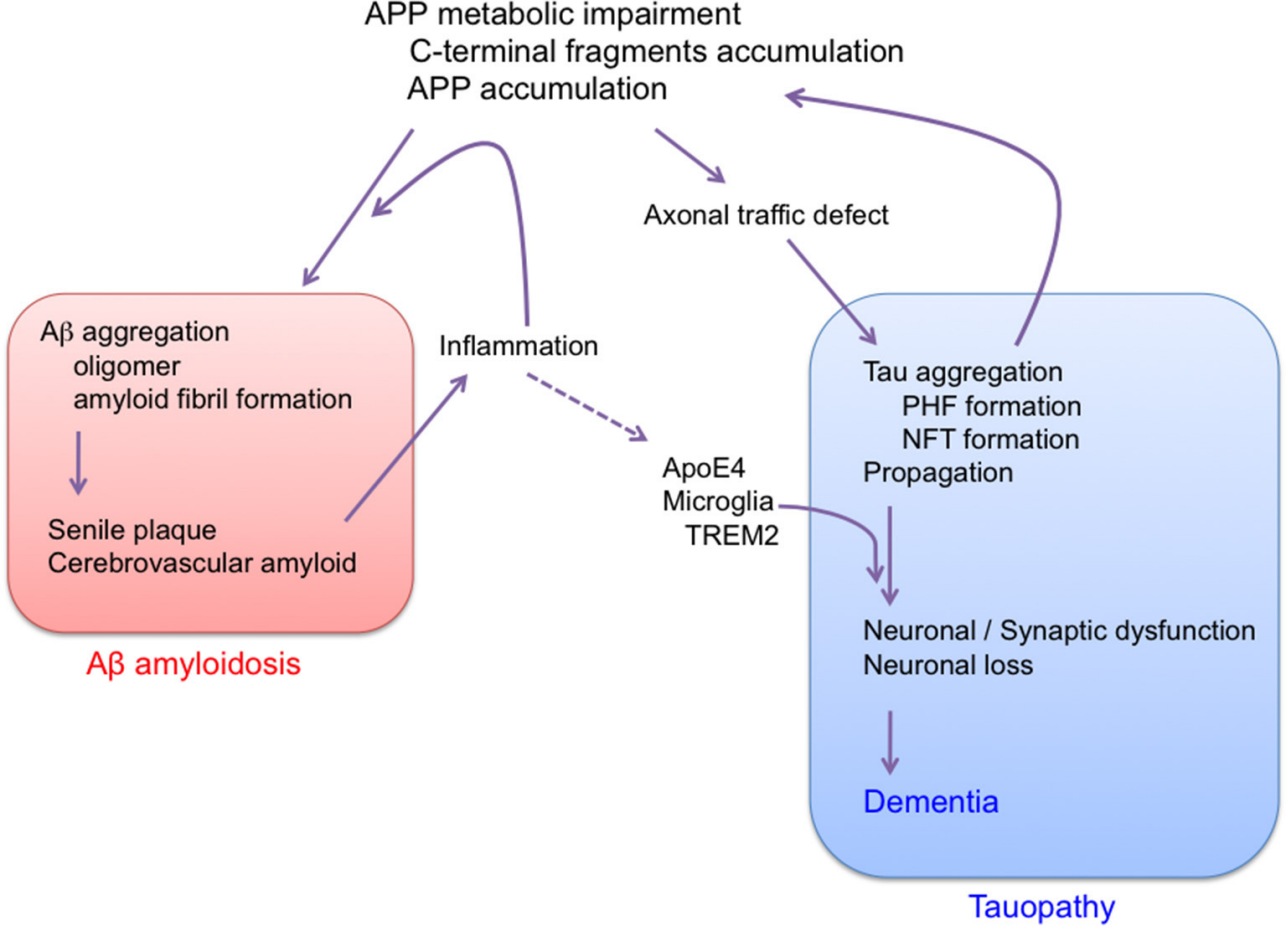
Proposed sequence of major pathogenic events leading to AD. Aβ amyloidosis and tau pathology are regarded as independent pathological events. AD is APP trigger tauopathy.

Overall, the results described above suggest that AD is a disorder that is triggered by impairment of APP metabolism, and that progresses through tau pathology (Figure 3). It is well-known that Aβ amyloidosis due to APP metabolic impairment leads to neuroinflammation, which may further affect the progression of tau pathology (Leyns and Holtzman, 2017). So far, there is no evidence that Aβ itself directly affects tau pathology. We cannot rule out the possibility that APP metabolic impairment and tau pathology might be initiated independently in sporadic AD. In any event, there is now convincing evidence that the main factor causing progression of AD is tau, not Aβ, and that Aβ amyloidosis and tau pathology should be regarded as independent pathological events. Indeed, it was recently shown that the AD risk factors ApoE4 and TREM2 are linked to tau pathology (Bemiller et al., 2017; Leyns et al., 2017; Shi et al., 2017). Moreover, the incidence of type 2 diabetes is increased in AD patients (Janson et al., 2004), and it was recently shown that tau protein is involved in the control of brain insulin signaling (Marciniak et al., 2017). Furthermore, the brains of patients with primary age-related tauopathy (PART) contain NFTs that are indistinguishable from those of AD, in the absence of Aβ amyloid plaques (Crary et al., 2014; Duyckaerts et al., 2015; Jellinger et al., 2015). Therefore, tau could contribute to the cognitive and metabolic alterations in patients with AD.

The unexpected failures of all trials of AD treatment candidate drugs targeting Aβ can easily be understood if the main factor causing progression of AD is tau, not Aβ. Indeed, it has already been reported that the suppression or deletion of tau has a profound protective effect against brain damage and neurological deficits (Rapoport et al., 2002; SantaCruz et al., 2005; Roberson et al., 2007; Miao et al., 2010; Shipton et al., 2011; Bi et al., 2017). Thus, suppression of tau production currently seems to be the most promising target for development of AD therapeutic drugs.

## Conclusion

The amyloid hypothesis has been the mainstream concept underlying AD research for over 20 years. However, reconsideration of APP and presenilin (PS) mutations in familial AD indicate that the trigger of AD is closely linked to impairments of APP metabolism and accumulation of APP C-terminal fragments, rather than Aβ production and Aβ amyloid formation. Furthermore, all attempts to develop Aβ-targeting drugs to treat AD have ended in failure and recent findings indicating that the main factor underlying the development and progression of AD is tau, not Aβ. Therefore, AD is a disorder that is triggered by impairment of APP metabolism, and progresses through tau pathology, not Aβ amyloid.

## Author contributions

FK and MH, data collection, literature review, manuscript writing.

### Conflict of interest statement

The authors declare that the research was conducted in the absence of any commercial or financial relationships that could be construed as a potential conflict of interest.
